# Urine ALCAM, PF4 and VCAM-1 Surpass Conventional Metrics in Identifying Nephritis Disease Activity in Childhood-Onset Systemic Lupus Erythematosus

**DOI:** 10.3389/fimmu.2022.885307

**Published:** 2022-05-26

**Authors:** Samar A. Soliman, Anam Haque, Kamala Vanarsa, Ting Zhang, Faten Ismail, Kyung Hyun Lee, Claudia Pedroza, Larry A. Greenbaum, Sherene Mason, M. John Hicks, Scott E. Wenderfer, Chandra Mohan

**Affiliations:** ^1^ Rheumatology and Rehabilitation Department, Faculty of Medicine, Minia University, Minia, Egypt; ^2^ Department of Biomedical Engineering, University of Houston, Houston TX, United States; ^3^ Center for Clinical Research and Evidence-Based Medicine, University of Texas Health Science Center at Houston, Houston, TX, United States; ^4^ Pediatric Nephrology, Emory University, Atlanta, GA, United States; ^5^ Connecticut Children’s Medical Center, University of Connecticut School of Medicine, Hartford, CT, United States; ^6^ Texas Children’s Hospital, Baylor College of Medicine, Houston, TX, United States

**Keywords:** ALCAM, VCAM 1, PF4, urine, biomarker, childhood-onset lupus

## Abstract

**Objectives:**

Serial kidney biopsy for repeat evaluation and monitoring of lupus nephritis (LN) in childhood-onset Systemic Lupus Erythematosus (cSLE) remains challenging, thus non-invasive biomarkers are needed. Here, we evaluate the performance of ten urine protein markers of diverse nature including cytokines, chemokines, and adhesion molecules in distinguishing disease activity in cSLE.

**Methods:**

Eighty-four pediatric patients meeting ≥4 ACR criteria for SLE were prospectively enrolled for urine assay of 10 protein markers normalized to urine creatinine, namely ALCAM, cystatin-C, hemopexin, KIM-1, MCP-1, NGAL, PF-4, Timp-1, TWEAK, and VCAM-1 by ELISA. Samples from active renal (LN) and active non-renal SLE patients were obtained prior to onset/escalation of immunosuppression. SLE disease activity was evaluated using SLEDAI-2000. 59 patients had clinically-active SLE (SLEDAI score ≥4 or having a flare), of whom 29 patients (34.5%) were classified as active renal, and 30 patients (35.7%) were active non-renal. Twenty-five healthy subjects were recruited as controls.

**Results:**

Urine concentrations of ALCAM, KIM-1, PF4 and VCAM-1 were significantly increased in active LN patients versus active non-renal SLE, inactive SLE and healthy controls. Five urine proteins differed significantly between 2 (hemopexin, NGAL, MCP1) or 3 (Cystatin-C, TWEAK) groups only, with the highest levels detected in active LN patients. Urine ALCAM, VCAM-1, PF4 and hemopexin correlated best with total SLEDAI as well as renal-SLEDAI scores (p < 0.05). Urine ALCAM, VCAM-1 and hemopexin outperformed conventional laboratory measures (anti-dsDNA, complement C3 and C4) in identifying concurrent SLE disease activity among patients (AUCs 0.75, 0.81, 0.81 respectively), while urine ALCAM, VCAM-1 and PF4 were the best discriminators of renal disease activity in cSLE (AUCs 0.83, 0.88, 0.78 respectively), surpassing conventional biomarkers, including proteinuria. Unsupervised Bayesian network analysis based on conditional probabilities re-affirmed urine ALCAM as being most predictive of active LN in cSLE patients.

**Conclusion:**

Urinary ALCAM, PF4, and VCAM-1 are potential biomarkers for predicting kidney disease activity in cSLE and hold potential as surrogate markers of nephritis flares in these patients.

## Introduction

Childhood-onset systemic lupus erythematosus (cSLE) is a complex, chronic, multisystem autoimmune disease with a significant impact on the affected child or adolescent under 18 years of age. Despite sharing similar pathogenesis with adult-onset SLE (aSLE), the clinical presentation of cSLE is generally more severe, with higher disease activity and damage, requiring more aggressive treatment (1–4). cSLE comprises approximately 15-20% of SLE cases, with a prevalence of 1.89-25.7 per 100,000 children and an annual incidence of less than 1 per 100,000 children, rendering it a rare disease in childhood, with considerably lower rates than adults. Accordingly, clinical research is more challenging, and evidence-based guidelines are lacking (5–8).

Compared to aSLE, cSLE exhibits higher frequencies of kidney, neuropsychiatric, and hematologic involvement (1, 4). Lupus nephritis (LN) continues to be a prominent source of morbidity and mortality in SLE, reported in 30-40% of cSLE patients (3–5, 8–11). LN is more frequent in Hispanics, and African descendants, showing higher levels of disease activity and risk of developing kidney failure compared to non-Hispanic whites (6). Proliferative lesions (focal or diffuse) are the most common biopsy finding in childhood LN, with approximately 10% of affected children progressing to kidney failure within 5 years (3).

Since LN treatment is commonly associated with significant side effects, clinical care must balance optimal control of inflammation and tissue injury with minimization of immunosuppressive therapy side effects. The absence of serologic and biochemical diagnostics that adequately indicate the type and extent of kidney inflammation is one of the obstacles to this approach (12). Currently utilized markers of SLE and LN disease activity, for example anti-double stranded DNA (ds-DNA), serum complement levels, creatinine, and urinary protein excretion have significant limitations. They are inconsistent at predicting approaching disease flares, which may start without any significant alteration in their levels (12, 13). Likewise, proteinuria and measures of kidney function such as serum creatinine lack specificity to lupus-related kidney inflammation and injury. Consequently, reliance on changes in proteinuria or serum creatinine as a marker of LN delays starting adequate therapy, and kidney biopsy remains the gold standard to distinguish between activity and chronicity of LN histopathology. As a result, there is a dire need for identifying biomarkers which reliably signify the degree, nature, and course of kidney inflammation in LN (14).

Urine is a promising body fluid for identifying LN-specific biomarkers. Several studies comparing levels of various biomarkers in serum and urine of active LN reported superiority of urine in predicting LN activity (12, 15–18). In the current study, we investigate the efficacy of a panel of 10 urinary proteins representing groups of molecules hypothesized to be implicated in the pathogenesis of lupus *via* diverse pathways, including cytokines or chemokines and their receptors [e.g., monocyte chemoattractant protein-1 (MCP-1), platelet factor-4 (PF4), and tumor necrosis factor-like weak inducer of apoptosis (TWEAK)], metalloproteinases inhibitors [e.g., tissue inhibitor of metalloproteinase-1 (TIMP-1)], cell adhesion molecules (CAMs) [e.g., activated leukocyte CAM (ALCAM) and vascular CAM-1 (VCAM-1)], acute phase reactant glycoproteins (e.g., hemopexin), and markers of kidney damage [e.g., cystatin-C, kidney injury molecule-1 (KIM-1) and lipocalin2/neutrophil gelatinase-associated lipocalin (NGAL)], as markers of disease flare in a well-phenotyped cSLE cohort. Moreover, these proteins have previously been implicated as biomarkers in adult patients with LN, as discussed below.

## Patients and Methods

### Patients

Eighty-four pediatric patients (≤18 years of age) fulfilling the revised 1997 classification criteria of the American College of Rheumatology (ACR) for SLE (19) were recruited into this study from the Pediatric Nephrology Research Consortium (PNRC) LN-Autoantibodies study cohort, with patients enrolled from pediatric clinics at Connecticut Children’s Medical Center, Texas Children’s Hospital (TCH), and Children’s Healthcare of Atlanta. Institutional review boards (IRBs) at Baylor College of Medicine (H-35050), the University of Connecticut, Emory University, and the University of Houston all gave their approval to the study. Based on good clinical practice and the Declaration of Helsinki, all recruited patients completed an IRB-approved informed consent form.

Prospectively, demographics, clinical characteristics and conservative metrics of disease activity, such as anti-dsDNA, C3 and C4 levels, serum creatinine levels, spot urine protein-to-creatinine ratio (uPCR), and eGFR (assessed by Bedside Schwartz equation) were collected. **Table 1** highlights demographics and clinical characteristics of all patients. Twenty-five healthy subjects of same sex and age were recruited as controls from TCH’s Gynecology and Adolescent Medicine Clinic.

**Table 1:** Patient demographics and clinical characteristics of the cSLE cohort.

Features	All SLE		Active Renal		Active Non-renal	Inactive SLE	Healthy Controls
**Number**	84		29		30	25	25
**Age**, mean ± SD	15.22 ± 2.7		15.1 ± 2.7		15.2 ± 2.6	15.4 ± 2.9	15.3 ± 1.85
**Females,** N(%)	73( 86.9)		27 (93.1)		26 (86.7)	20(80)	25 (100)
**Race**							
Hispanic, N(%)	46 (54.8)		16		16	14	14
African American, N(%)	23 (27.4)		8		10	5	6
Caucasian, N(%)	8 (9.5)		2		2	4	2
Asian, N(%)	5 (5.9)		1		2	2	3
Mixed, N(%)	2 (2.4)		2		0	0	0
**SLE disease duration,** median (IQR), mos	6.8 (0.2-30)		0.3 (0.1-2.6)		0.7 (0.2-17)	28 (16-55)	–
**SLEDAI,** median (IQR)	4 (0-11)		12 (8-22)		4 (4-6)	0 (0-2)	–
**Historic SLE Manifestations** (%)							
Neuropsychiatric	12%		18%		10%	9%	–
Musculoskeletal	60%		64%		57%	56%	–
Kidney disorder	61%		100%		17%	58%	–
Mucocutanous	52%		57%		58%	38%	–
Serositis	22%		32%		14%	21%	–
Hematological	83%		86%		72%	85%	–
Features of renal disease*							
Serum creatinine, mean (SD), mg/dL			0.78 ± 0.11		0.55 ± 0.03	0.62 ± 0.02	–
eGFR, mean (SD), ml/min/1.73m^2^			119.1 ± 18.2		115.2 ± 3.9	102 ± 3.7	–
urine PCR, mean (SD), mg/mg			3.29 ± 0.95		0.12 ± 0.08	0.11 ± 0.09	–
Renal SLEDAI score, median (IQR)†			8 (4-12)		0	0	–+

eGFR, estimated glomerular filtration rate; IQR, Interquartile range; SLEDAI, Systemic Lupus Erythematosus Disease Activity Index; IQR, Interquartile range; PCR, Protein Creatinine ratio.

†: Range 0-16; 0 = inactive LN, *Healthy controls did not have proteinuria, as determined using a negative urine dipstick.

### Assessment of SLE Disease Activity and Flares

In the LN-autoantibodies study, enrolled patients were either incident patients who had their samples taken before starting immunosuppression, prevalent patients who had a recent lupus flare (before escalating immunosuppression), or prevalent patients who were in remission (on or off immunosuppression). SLEDAI-2000, an established index in research and clinical practice was used to evaluate SLE disease activity (20). Clinical LN activity was weighed using the renal domain scores of SLEDAI (range 0–16; 0 = inactive LN). Patients were divided into three groups at the time of enrollment: active renal SLE (LN, patients with a renal SLEDAI score of 4 or higher), active non-renal SLE (patients with active symptoms or organ involvement but a renal SLEDAI of 0), and inactive SLE (patients with a total clinical SLEDAI of 0, asymptomatic with no findings of organ activity, subclinical hypocomplementemia, and/or elevated autoantibodies allowed). The Systemic Lupus International Collaborating Clinics (SLICC)/ACR Damage Index (SDI) (range 0–47; 0=no SLE damage) was used to assess disease damage (21).

### Urine Biomarkers Assays

Prior to batch processing, urine samples were prepared, aliquoted, and frozen at -80°C before 2 hours of collection. Only one aliquot was recovered for each experiment to avoid repeated freeze/thaw cycles. Urine levels of ALCAM, cystatin-C, hemopexin, KIM-1, MCP-1, NGAL, PF-4, Timp-1, TWEAK, and VCAM-1 were measured by means of a human enzyme-linked immunosorbent assay (ELISA) kit. All biomarkers were tested using ELISA kits from R&D Systems (Minneapolis, Minnesota, USA), except for hemopexin (pre-coated ELISA kit from Immunology Consultants, Portland, OR, USA) according to the manufacturer’s manual. A microplate reader ELX808 (BioTek Instruments, Winooski, VT) was used to detect optical densities at 450 nm, and sample concentrations were estimated using a standard curve. All measurements were double-checked. Urine samples were diluted 1:2 for ALCAM and KIM-1, 1:5 (MCP-1, NGAL, PF-4, TIMP-1 and TWEAK), 1:50 (hemopexin and cystatin-C) and 1:100 for VCAM-1. All tested dilutions in the initial screening cohort prior to testing in the validation cohort are presented in **Supplementary Table 2**. Urine creatinine was used to standardize the results of urinary protein markers. Biomarker assay performers and readers were blinded to patient groups and clinical information.

### Renal Histology

Patients were registered in this experiment if a random spot urine sample was obtainable within 1 week of the kidney biopsy. The renal histopathologic features of the active renal group were evaluated by doing a kidney biopsy assessed by one pediatric nephropathologist, blinded to the patients’ biomarker expression data. The International Society of Nephrology/Renal Pathology Society (ISN/RPS) criteria were used to determine LN classification, histologic features of active inflammation, and features of chronicity or degenerative damage associated with LN (22). Biopsy activity and chronicity indices (AI, CI, respectively) were employed to assess biopsy activity and chronicity in accordance with the National Institute of Health’s LN guidelines (23). Activity and chronicity features are given numeric values, which are further used to estimate the AI score (range 0-24; 0= no LN activity) and CI score (range 0-12; 0= no LN chronicity) (23), with AI and CI scores of ≥7 and ≥4, respectively considered as poor prognostic risk factors for LN outcomes upon long-term follow-up (24).

### Data Analysis

For interval and ordinal data, means± standard deviations (SD), and ranges were calculated; for categorical variables, frequencies and percentages were calculated. The mean-standard error of the mean was used to express continuous variables (SEM). To check for data normality, the Kolmogorov–Smirnov and Shapiro–Wilk tests were utilized. If the data was not normally distributed, the results were reported as medians and interquartile ranges (IQR). The non-parametric Kruskal-Wallis H (continuous variables) or chi-square (categorical variables) tests were used to compare values between groups. For correlation analysis of continuous and regularly distributed data, Pearson’s correlation coefficient was utilized. The nonparametric Spearman’s correlation coefficient was used otherwise. Rho values of 0.2–0.4 were rated mild; 0.4–0.6 were considered modest; and >0.6 were considered high. Statistical significance was defined as a two-tailed P-value of less than 0.05.

Receiver operating characteristic curve (ROC) analysis was used to examine the diagnostic accuracy of each biomarker as well as traditional SLE indicators, and the associated area under the curve (AUC; range 0–1) was obtained. The sensitivity, specificity, positive and negative predictive values, and ideal cut-off values were all determined using ROC analysis. GraphPad Prism v.6.0 was used for all statistical analyses (GraphPad, San Diego, CA, USA).

To identify the multi-marker panel of proteins that best discriminate groups of subjects, the predictive projection feature selection technique (25, 26) was implemented using the projpred package in R (version 4.0.3) (27). Model selection was conducted based on a model with the best predictive power (reference model) to locate a simpler model with a smaller number of proteins that maintains comparable prediction performance compared to the reference model (predictive projection). This selection process consisted of two main steps. First, we fitted a Bayesian regularized logistic regression model with horseshoe prior (28, 29), including all 8 proteins as a reference model. Second, we searched for a projected submodel with (at most) 5 proteins that minimized the Kullback-Leibler divergence from the posterior distribution of the reference model to that of the projected model. The selected submodel exhibited a similar predictive performance determined by the mean log predictive density and the mean squared error. Both performance metrics, along with area under the curve (AUC) and prediction accuracy, were evaluated through leave-one-out cross validation (LOOCV) to bypass potential problems of overfitting. The selected proteins of one model and its model performance metrics were compared to those of the counterpart model with adjustment for age, gender (male and female) and race (Caucasian and other) to account for potential confounder effects from these variables.

## Results

### Study Population Characteristics and Histopathologic Features of Active Lupus Nephritis Subjects

A total of 84 patients with SLE (86.9% female) were enrolled in this study. Their mean age was 15.2 ± 2.7 years. The patients’ median SLEDAI score was 4, with scores ranging from 0 to 33. According to their SLEDAI results, 29 patients (34.5%) had active renal disease, 30 patients (35.7%) had active non-renal disease, and 25 patients (29.8%) had clinically inactive SLE. All patients had their SLE disease damage measured using the SLICC damage index, which was classified as 0 or 1 at the time of enrollment. As controls, 25 healthy subjects (all females, mean age 15.31.8) were included.

Whether renal or non-renal, all active SLE patients were sampled before initiating immunosuppression apart from oral prednisolone or intravenous (IV) methylprednisolone. Low dose immunosuppression, in the form of prednisone (59%, median dose 2.5mg/day), hydroxychloroquine (84%), azathioprine (25%), mycophenolate mofetil (50%), or methotrexate (9%) were used as maintenance therapy for inactive SLE patients. For inactive patients who had rituximab (63%), samples were collected at a median of 437 days following the last dosage (IQR 215-716 days). Also, those who had IV methylprednisolone were sampled on average 455 days following their previous dosage (78%) (IQR 387-716 days).

Comparing patients with active renal disease to those with active non-renal and inactive SLE disease, their total SLEDAI scores were significantly higher (median 12; range 4-33) (P<0.0001) (**Table 1**). The median renal SLEDAI score was 8 among the 29 individuals with active LN (range 4-16). The uPCR concentrations ranged from 0.08 to 21.5 mg/mg, with significant increase in active renal patients compared to active non-renal and inactive SLE (P<0.0001). However, both serum creatinine and eGFR did not exhibit any significant differences among the three patient groups (P= 0.1235 and 0.102, respectively). In 15 (51.7%), 20 (69%), and 12 (41.4%) individuals, respectively, pyuria, hematuria, and active urinary casts were found. In twenty-three (79.3%) of the patients, a kidney biopsy was conducted. None of them revealed ISN/RPS class IV LN isolated. ISN/RPS classes VI and V were identified in 6 (26%) patients each, ISN/RPS class III in 3 (13.6%) patients, and mixed class LN (III+V or IV+V) in 5 (21.7%) patients. The proliferative LN subgroup (N = 14) included patients with ISN/RPS class III/IV± V, while the non-proliferative LN subgroup (N = 9) included those with other histological classes of nephritis (ISN/RPS I/II/pure V). In the same setting, histopathologic aspects of LN activity and chronicity were assessed simultaneously (**Table 2**), with a median biopsy activity score of 4 (range 0-17) and chronicity index of 0 (range 0-3).

**Table 2:** Histologic features of the active lupus nephritis patients.

ISN/RPS classification (n=23)	
Class I (Minimal mesangial LN), N (%) Class II (Mesangial proliferative LN), N (%) Class III (Focal LN), N (%) Class IV (Diffuse LN), N (%) Class V (Membranous LN) (pure), N (%)	2 (8.7) 1 (4.3) 3 (13.6) 6 (26.1) 6 (26.1)
Mixed class III/IV and V, N (%)	5 (21.7)
**Histologic features (n = 23)**	
**Activity Index, median (IQR)^§^**	*4 (0-17)*
Endocapillary proliferation score >0, N (%)	12 (54.5)
Glomerular WBC infiltration score >0, N (%)	9 (40.9)
Hyaline deposits score >0, N (%)	7 (31.8)
Karyorrhexis score >0, N (%)	5 (22.7)
Cellular crescents score >0, N (%)	5 (22.7)
Interstitial inflammation score >0, N (%)	9 (40.9)
No active lesions noted, N (%)	5 (22.7)
***Chronicity Index, median (IQR)*** *¶*	*0 (0-3)*
Glomerulosclerosis score >0, N (%)	6 (27.3)
Fibrous crescents score >0, N (%)	0
Tubular atrophy and interstitial fibrosis scores >0, N (%)	7 (31.8)
No chronicity noted, N (%)	15 (65.2)

SLEDAI, Systemic Lupus Erythematosus Disease Activity Index; ISN/RPS, International Society of Nephrology/Renal Pathology Society.

§: Range 0-24; 0 = no LN activity features, ¶: Range 0-12; 0 = no LN chronic change.

### Urine Levels of Assayed Protein Markers

**Figure 1:**
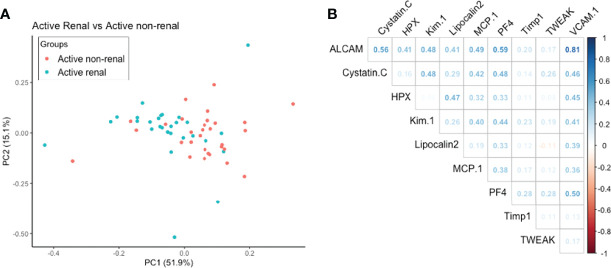
**(A)** Principal Component Analysis aiming to discriminate active renal SLE from active non-renal SLE using 10 urine proteins assayed in the cSLE cohort. Together the first 2 components accounted for 67% of the variance between these two disease groups. **(B)** Correlation of levels of assayed urine protein markers with each other. Correlation coefficient between the 10 markers is represented by density of blue (for positive correlation) or red color (for negative correlation).

**Figure 2:**
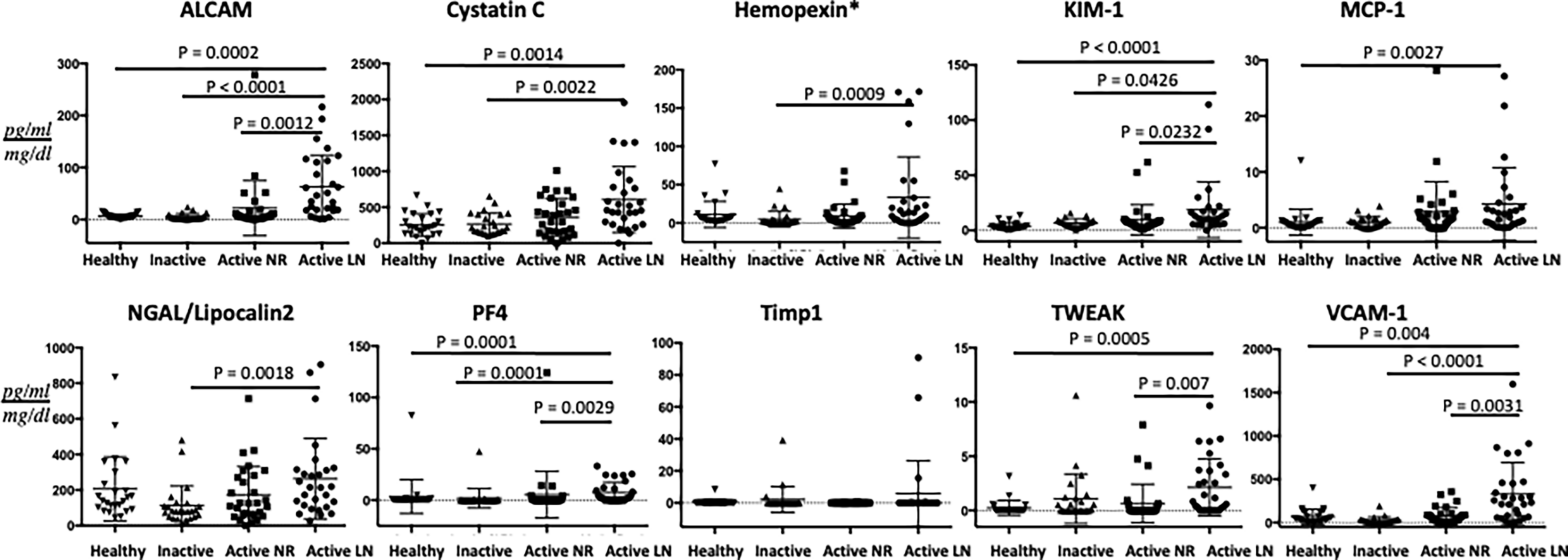
Urine concentrations of the ten assayed proteins in the cSLE cohort. The concentrations of the 10 proteins investigated are shown on the Y-axes. The four groupings are represented by the X-axes (29 active renal; 30 active non-renal; 25 inactive cSLE and 25 healthy controls). Means and SE (error bars) are shown. Only comparisons achieving statistical significance are shown with *P*-values. All biomarkers’ values are in pg/ml, normalized to urine Cr, except urine Hemopexin (*) which is expressed as ng/ml normalized to urine Cr.

As a group, the 10 urine biomarker proteins showed ability to discriminate active LN patients from active non-renal SLE patients, based on principal component analysis, as illustrated in **Figure 1A**. Among the 10 assayed biomarkers, particularly noteworthy was the high correlation of urine ALCAM with VCAM1 and PF4. Furthermore, we examined the performance of each individual marker (**Figure 2**). Urine levels of ALCAM, KIM-1, PF4 and VCAM-1 were significantly increased in active LN patients versus all other groups of patients: active non-renal, inactive SLE and controls. However, no significant difference in urine Timp1 concentrations among the 4 groups was detected. Urine concentrations of cystatin-C and TWEAK were significantly higher in patients with active renal disease than in healthy controls (P= 0.0014, 0.0005, respectively). In addition, urine cystatin-C levels were significantly increased in active LN than inactive SLE patients (P=0.0022), while urine TWEAK levels exhibited significant increase in active LN than active non-renal patients (P=0.007). Urine levels of hemopexin and lipocalin2/NGAL were only significantly different between active renal and inactive SLE patients (P=0.009, 0.0018, respectively), whereas urine MCP-1 levels only showed significant difference between active renal and healthy controls (P=0.0027).

Among the active renal lupus patients with a concurrent kidney biopsy, urine hemopexin and KIM-1 levels showed an increase of approximately 4- and 2-folds in the urine of patients with proliferative LN classes in contrast to non-proliferative LN classes. These differences, however, were not statistically significant. Non-significant increases in urine ALCAM, cystatin-C, MCP-1, NGAL, PF4 and VCAM-1 (47%, 48%, 19%, 86%, 15% and 36%) in patients with proliferative LN classes were detected as well. Urine levels of Timp-1 and TWEAK were comparable in proliferative and non-proliferative LN patients. The proliferative LN subgroup had significantly lower serum complement C3 levels (37.1± 4.3 vs. 77.5± 15.1 mg/dl, P = 0.02). Patients with proliferative LN classes exhibited significantly greater levels of pyuria (11 vs. 1, P = 0.003) and hematuria (14 vs. 4, P = 0.003) than those with non-proliferative classes. Consequently, SLEDAI as well as the renal domain of SLEDAI scores were significantly higher in proliferative LN patients [median (IQR) 21(8-33) vs. 12(4-27), P=0.018] and [median (IQR) 12(4-16) vs. 4(4-8), P= 0.012], respectively. The renal biopsy activity index was significantly higher in proliferative LN [median (IQR) 10 (4-12) vs. 0 (0-1), P <0.0001], while the biopsy chronicity index showed no significant difference.

### Biomarkers’ Performance in Distinguishing Global and Renal Disease Activity in SLE Patients

**Figure 3:**
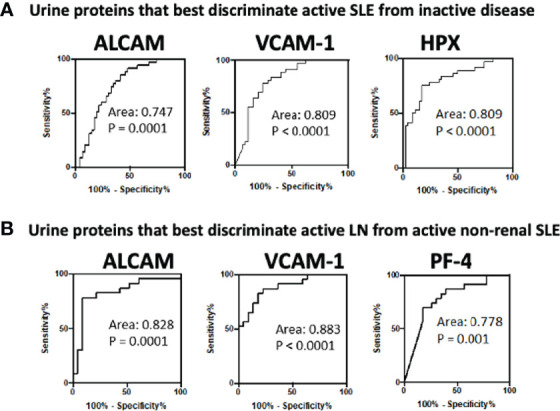
Receiver operating characteristic (ROC) curves for the best 3 urine biomarkers in differentiating **(A)** active cSLE (renal and non-renal) from inactive cSLE patients and **(B)** active renal from active non-renal cSLE patients.

ROC analysis was conducted to evaluate the effectiveness of the ten urine biomarkers in distinguishing active renal from active non-renal and active SLE from inactive SLE participants, in comparison to serum anti-dsDNA and low C3 levels (**Table 3**). Urine VCAM-1 and hemopexin were the most discriminatory proteins (AUC 0.81 both, P =0.009 and <0.0001, respectively) for distinguishing disease activity in cSLE independent of the end organ affected, while urine ALCAM showed good performance (AUC 0.75, P =0.0001). Importantly, urine VCAM-1 and ALCAM showed excellent ability in discriminating renal disease activity among active SLE patients (with sensitivity and specificity vales ranging from 78-92%), whereas urine PF4 displayed good performance in this respect (AUC 0.778, P =0.001), as indicated in **Table 3** and **Figure 3**. All of the biomarkers listed above outperformed anti-dsDNA in discriminating active (renal) cSLE from other cSLE patients.

**Table 3:** Performance of the protein markers in differentiating SLE and LN disease activity.

Markers	Cut-off	AUC (95%CI)	Specificity	Sensitivity	P
**Active SLE (active renal & active non-renal) Vs. Inactive SLE**					
**VCAM-1**	> 38.5 pg/ng	0.81(0.714 – 0.904)	60.87	74.29	0.009*
**Hemopexin**	>2.68 ng/ng	0.81 (0.713-0.905)	82.6	75	<0.0001*
**ALCAM**	>14.7 pg/ng	0.747 (0.64-0.85)	58.7	85.7	0.0001*
**MCP-1**	>1.402 pg/ng	0.669 (0.549-0.788)	60.87	74.29	0.009*
**NGAL**	>127.8 pg/ng	0.662 (0.54-0.783)	63	77.14	0.0125*
**PF4**	>0.182 pg/ng	0.654 (0.535-0.773)	58.7	73.5	0.0188*
**Cystatin-C**	>423 pg/ng	0.603 (0.478-0.726)	54.38	77.14	0.1158
**KIM-1**	>8.93 pg/ng	0.59 (0.465-0.714)	50	80	0.1669
**Tweak**	>2.517 pg/ng	0.588 (0.464-0.711)	28.3	88.9	0.172
**Timp-1**	>0.125 pg/ng	0.536 (0.408-0.663)	93.5	13.9	0.578
**Anti-dsDNA**	>30 IU/ml	0.67 (0.55-0.79)	59	77	0.01*
**Active renal Vs. active non-renal SLE**					
**VCAM-1**	>125.7 pg/ng	0.883 (0.787-0.979)	81.8	82.6	<0.0001*
**ALCAM**	>16.3 pg/ng	0.828 (0.698-0.958)	91.3	78.3	0.0001*
**PF4**	>0.197 pg/ng	0.778 (0.641-0.916)	82.6	69.57	0.001*
**Tweak**	>0.1625 pg/ng	0.746 (0.599-0.894)	78.3	73.9	0.004*
**KIM-1**	>9.066 pg/ng	0.735 (0.586-0.885)	73.9	78.3	0.006*
**Cystatin-C**	>427.1 pg/ng	0.728 (0.582-0.876)	69.57	69.57	0.008*
**Hemopexin**	>8.78 ng/ng	0.720 (0.568-0.872)	73.9	73.9	0.0105*
**MCP-1**	>2.448 pg/ng	0.709 (0.56-0.859)	60.8	78.3	0.015*
**NGAL**	>59.5 pg/ng	0.646 (0.487-0.805)	95.6	30.4	0.088
**Timp-1**	>0.242 pg/ng	0.565 (0.398-0.733)	13.04	100	0.448
**Anti-dsDNA**	>120 IU/ml	0.55 (0.39-0.71)	48	69	0.485

AUC, area under the curve; SLE, systemic lupus erythematosus; CI, confidence interval. Biomarkers are listed in rank order by AUC values from highest to lowest.

*means statistically significant.

We also constructed multi-marker panels after adjusting for demographic variables, using predictive projection feature selection. As shown in **Supplementary Table 1**, the multi-marker panel that best distinguished active LN from active non-renal SLE was a panel composed of ALCAM and PF4 (ROC AUC = 0.71, Accuracy = 0.76, after adjusting for demographic variables), while urine ALCAM alone outperformed all multi-marker panels in distinguishing active SLE from inactive SLE.

### Correlation of Urine Biomarkers With SLE Disease Activity and Renal Parameters

**Figure 4:**
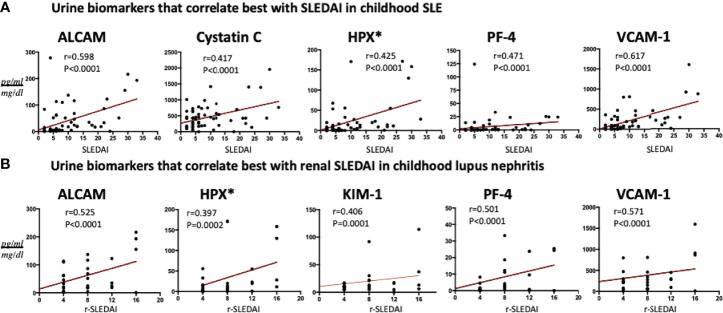
Association of the best 5 urine protein markers with **(A)** SLEDAI in all SLE patients and **(B)** renal-SLEDAI in active renal SLE patients. All biomarkers’ values are in pg/ml, normalized to urine Cr, except urine Hemopexin (*) which is expressed as ng/ml normalized to urine Cr.

In SLE patients (N = 84), urine ALCAM and VCAM-1 showed a strong significant correlation with total SLEDAI scores (**Figure 4A**), while urine PF4, hemopexin and cystatin-C revealed good correlations with SLEDAI (r 0.47, P<0.0001; r 0.43, P<0.0001; r 0.42, P<0.0001), respectively. Among active lupus nephritis patients (**Figure 4B**), urine VCAM-1 (r 0.57, P<0.0001), ALCAM (r 0.53, P<0.0001) and PF4 (r 0.50, P<0.0001) exhibited the best correlations with renal SLEDAI.

**Figure 5:**
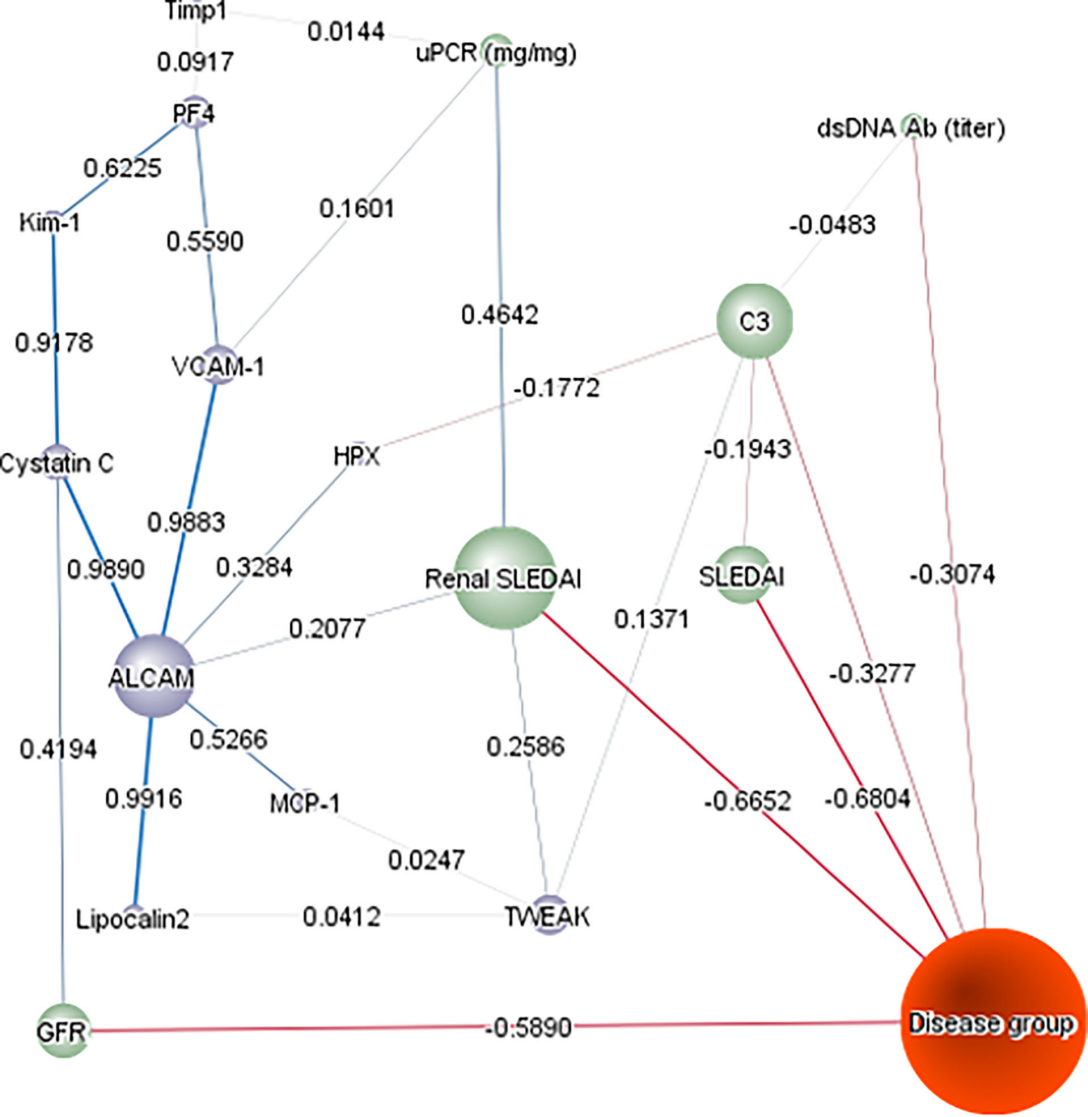
Bayesian Network Analysis. The levels of the 10 urine proteins in the cSLE cohort and their relevant clinical characteristics were analyzed using Bayesian network analysis using BayesiaLab. The presented network was assembled in an unsupervised manner, using the EQ algorithm and a structural coefficient of 0.4. The circular nodes making up the Bayesian Network denote the variables of interest, including urine protein markers (purple-colored), clinical indices (green-colored), and disease group (inactive/active non-renal, active renal; colored orange). The “node force” is denoted by the size of each node, reflecting its effect on other nodes in the network, according to conditional probabilities. The informational or causal dependencies among the variables are represented by the links (arcs) that connect the nodes, including the correlation coefficients between adjacent nodes (as stated), with the thickness of the link being proportional to the correlation coefficient.

We then subjected the 10 assayed urine proteins, ethnicity, and various clinical metrics to an unsupervised Bayesian network analysis (**Figure 5**), to investigate the interdependencies of all changing variables in a model and how they relate to one another, using probability distributions. As predicted, rSLEDAI was strongly linked to proteinuria and the disease group, offering independent validation of this unsupervised approach. Likewise, the close association/correlation of urine Cystatin C with eGFR also supports the validity of this methodology. This independent analysis re-affirmed urine ALCAM as the biomarker having the greatest impact on this complex, based on its “node force”, being proportional to the size of each node, correlating strongly with urine VCAM-1, Kim1/Cystatin-C and Lipocalin-2, all of which had weaker impacts on this network.

### Correlation of Urine Biomarkers With Biopsy Activity and Chronicity Histopathologic Features

**Figure 6:**
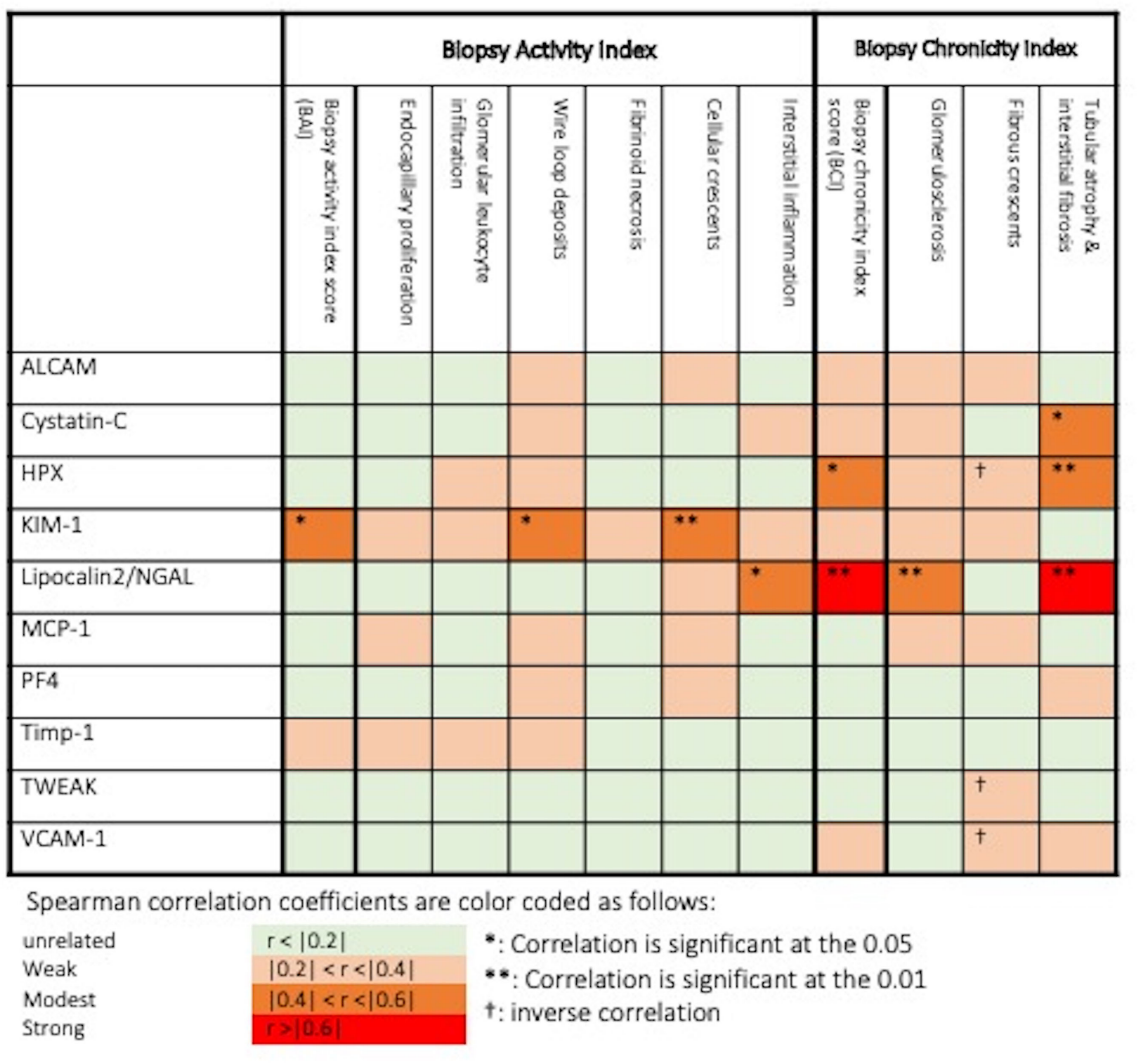
Association of urine biomarkers levels and histopathologic features in biopsied active lupus nephritis patients. *: Correlation is significant at p = 0.05, **: Correlation is significant at p = 0.01, †: inverse correlation. Spearman correlation coefficients are color coded as follows: r <0.2: unrelated, 0.2 ≤ r <0.4: weak, 0.4 ≤ r < 0.6: modest, r ≥ 0.6: strong.

We further investigated the correlation of the assayed biomarkers with histopathologic features of renal biopsy activity and chronicity indices among active LN patients who had concurrent kidney biopsies (N=23). As shown in **Figure 6**, urine KIM-1 was the only protein to correlate significantly with AI score, as well as two activity features, while urine Lipocalin2/NGAL and HPX significantly correlated with the CI scores. The former two proteins also correlated with different chronicity features, in addition to cystatin-C, which correlated with tubular atrophy and interstitial fibrosis.

## Discussion

In the present study, we have investigated the performance of ten urine proteins as potential biomarkers for lupus nephritis in a cSLE cohort. We demonstrated that all tested urinary proteins, except for Timp-1, were elevated in active LN patients. Urine ALCAM, KIM-1, PF4 and VCAM-1 were significantly increased in active LN patients in comparison to active non-renal, inactive SLE and controls. Urine ALCAM, VCAM-1, PF4 and hemopexin exhibited the best correlations with total SLEDAI as well as renal-SLEDAI scores. Urine ALCAM, VCAM-1 and hemopexin surpassed conventional laboratory metrics (anti-dsDNA, C3 and C4) in detecting clinical disease activity among cSLE patients, whereas ALCAM, VCAM-1 and PF4 levels were the best discriminators of renal disease activity in cSLE patients, outperforming conventional biomarkers including proteinuria. Based on the correlation analysis (**Figure 1B**) and the unsupervised Bayesian network analysis (**Figure 5**), urine ALCAM is likely driving the biomarker potential of VCAM1 and PF4. Indeed, Bayesian analysis also identified urine ALCAM as a driving factor in dictating the expression profiles of other urine markers in aSLE (30).

ALCAM, also known as cluster of differentiation-166 (CD-166) is a cell adhesion glycoprotein that is highly expressed on antigen-presenting cells and shows a fundamental role in mediating immune cell adhesion and migration, co-stimulation of T-cells and sustaining T cell activation. The role of ALCAM as a biomarker for inflammation, angiogenesis, diagnosis, prognosis, and treatment response in various cancers has been established (31). In diabetic nephropathy, serum concentrations of ALCAM as well as its expression in kidney tissue were significantly elevated and upregulated in glomeruli and tubules (32). ALCAM expression was also up-regulated in the glomeruli and tubules of MRL*/lpr* lupus-like murine model (33). In recent high-throughput proteomic approaches, urine ALCAM showed promise in predicting LN activity in SLE patients (30). Further validation in two aSLE cohorts confirmed the higher urine ALCAM levels with significant correlations with total and renal SLEDAI scores (34, 35).

The current study is the first to evaluate the performance of urine ALCAM in a cSLE cohort. In addition to being significantly increased in active LN patients, urine ALCAM correlated significantly with total and renal SLEDAI score and exhibited excellent ability to distinguish cSLE patients with active renal disease. Likewise, all multi-marker panels that exhibited outstanding diagnostic performance in distinguishing active (renal) cSLE included urine ALCAM. However, in contrast to findings in aSLE, urine ALCAM levels were not associated with proliferative LN or with renal pathology AI or CI, possibly due to the limited sample size with concurrent renal biopsies. Alternatively, this might be an indication that the molecular determinants of clinical disease activity and renal disease activity in cSLE may be distinct.

VCAM-1 or CD106 is a widely expressed cell adhesion molecule in peripheral circulation being expressed mainly in endothelial cells and glomerular parietal epithelial cells (36). Several studies have shown elevated and strongly correlated serum and urinary VCAM-1 levels with LN activity and severity (14, 18, 34, 37–40). Moreover, some studies reported its association with proliferative LN classes (39, 40), as well as its reduction following treatment (38). In resonance with the studies in aSLE, urine VCAM-1 was significantly elevated in active LN patients versus active non-renal, inactive SLE and healthy controls in our cohort. Urine VCAM-1 was highly predictive of SLE disease activity (when compared with inactive SLE, AUC 0.81) and more specifically with LN disease activity (when compared with active non-renal SLE patients, AUC 0.88). Furthermore, among the ten tested biomarkers, urine ALCAM and VCAM-1 revealed the best correlations with total and renal SLEDAI scores, although they did not reflect concurrent renal pathology activity, as discussed above. Two previous studies explored serum (41) and urine (42) VCAM-1 levels in a pediatric SLE, corroborating the present findings.

Another promising biomarker of SLE and LN is PF4, an anti-angiogenic chemokine functioning *via* an integrin-dependent mechanism to regulate angiogenesis. Anti-fibrotic cytokines (e.g., interferon- γ) are inhibited by PF4, while pro-fibrotic cytokines are promoted (e.g., IL-4 and IL-13). It also boosts the growth of regulatory T cells (43). Additionally, the roles of PF4 in cancer, atherosclerosis, and heparin-induced thrombocytopenia are well-established (44–46). Serum PF4 levels were found to be elevated in systemic sclerosis (47), as well as in the plasma of antiphospholipid syndrome (APS) patients (48), suggesting its role in the pathogenesis of these disorders.

Recent reports (12, 39, 43) have verified urinary PF4 as a promising biomarker distinguishing active LN adult patients and correlating with biopsy activity changes. Consistent with these findings, the current study found urinary PF4 levels to exhibit highest values in active LN patients compared to other SLE groups as well as controls. Additionally, the ability of the biomarker in distinguishing active LN from active non-renal SLE was “very good”. Furthermore, PF4 levels were among best biomarkers that correlated with renal SLEDAI as well as total SLEDAI scores. It showed good correlations with urine ALCAM and VCAM-1 concentrations, supporting the established link between VCAM-1 and PF4/CXCL4 and their receptors *via* the crosstalk between neutrophils and bone marrow endothelial cells (49). Similar interactions between these molecules may in part explain the coordinated elevations in these proteins in cSLE. Additional studies in aSLE cohorts provide further support for these proteins, endorsing their role as biomarkers for early detection of LN flare-ups and as potential therapeutic targets in LN. These include studies focusing on these biomarkers highlighting their potential utility in serial biomarker tracking, predicting clinical and pathological activity in LN, and biomarker-directed therapeutic targeting (50–52).

A strength of the present study was the identification of novel urine biomarkers for cSLE and childhood LN activity that had better diagnostic capacity than traditional kidney injury markers (e.g., Cystatin-C and KIM-1). Serum cystatin-C has been recommended as a promising marker of GFR, useful for estimating kidney function in both acute and chronic kidney dysfunction (53, 54). Indeed, its association with eGFR was re-confirmed in the unsupervised Bayesian network analysis. KIM-1 is an immunoglobulin domain and mucin domain-1 bearing protein induced in damaged tubular epithelial cells and is related to interstitial fibrosis and kidney inflammation (55). In our study, urinary cystatin-C and KIM-1 showed significant increases in active LN, and urinary KIM-1 was the only biomarker to correlate significantly with the renal pathology AI score, due to its correlation with cellular crescents and wire loops, an indicator of immune deposition.

The study’s limitations included the relatively low number of patients, and its cross-sectional design which prevented authors from interrogating biomarkers for prognosis or recording changes in disease activity over time, without the confounding effects of subject to subject variation. Pure class V LN, which behaves quite differently from proliferative (Class III or IV) lesions, were seen in a high proportion of the active LN subjects examined in this study. As a result, the Activity Index on renal biopsy in this group is rather low, at 4 points. Accordingly, proteinuria in the nephrotic range was the primary presenting feature in these patients, without significant inflammatory infiltrates. Future biomarker studies will have to include larger numbers of LN patients with proliferative renal disease.

To the best of our knowledge, this is the first study to assess the role of urinary ALCAM and PF4 in a cSLE cohort. Urine ALCAM and PF4 appear to have the greatest promise as SLE and lupus nephritis activity indicators, outperforming conventional markers in distinguishing active SLE and LN patients. Urine VCAM-1 and HPX levels have already been studied in many aSLE and cSLE cohorts, confirming earlier findings. Further longitudinal research is needed to confirm the performance of these urine proteins as disease flare predictors compared to traditional markers, as well as to see if combining these urine protein markers with anti-dsDNA and complement levels might offer improved sensitivity and specificity profiles in predicting early SLE relapse.

## Data Availability Statement

The LN-autoantibodies clinical dataset and paired urine samples are available upon request, in accordance with thePediatric Nephrology Research Consortium policies (https://pnrconsortium.org/). Anybiomarker data generated and/or analyzed during the current study that were not includedin this published article are available from the corresponding authors upon reasonable request.

## Ethics Statement

The studies involving human participants were reviewed and approved by The institutional review boards (IRBs) at Baylor College of Medicine (H-35050), the University of Connecticut, Emory University, and the University of Houston. Based on good clinical practice and the Declaration of Helsinki, written informed consent to participate in this study was provided by the participants’ legal guardian/next of kin.

## Author Contributions

SS, AH, KV, and TZ performed the experiments. KL, CP, and FI performed the data analyses. MJH performed all pathology analyses. LG, SM, and SW provided patient samples. SW and CM designed the studies and reviewed all data. SS, SW, and CM wrote the manuscript. All authors reviewed the manuscript and concurred with the findings. All authors contributed to the article and approved the submitted version.

## Funding

This work is supported by NIH R01 AR074096.

## Conflict of Interest

The authors declare that the research was conducted in the absence of any commercial or financial relationships that could be construed as a potential conflict of interest.

## Publisher’s Note

All claims expressed in this article are solely those of the authors and do not necessarily represent those of their affiliated organizations, or those of the publisher, the editors and the reviewers. Any product that may be evaluated in this article, or claim that may be made by its manufacturer, is not guaranteed or endorsed by the publisher.
