# Supplementary material for: Urine ALCAM, PF4 and VCAM-1 Surpass Conventional Metrics in Identifying Nephritis Disease Activity in Childhood-Onset Systemic Lupus Erythematosus
Source: Front Immunol. 2022 May 26;13:885307. doi: 10.3389/fimmu.2022.885307 (PMC9204340; doi:10.3389/fimmu.2022.885307)
Supplement: Supplementary file 1 [file Table_1.docx]

**Supplementary Table 1:**

|  | **With Adjustment** | **Without Adjustment** |
| --- | --- | --- |
|  | **Horseshoe prior** | **Horseshoe prior** |
| **Active renal LN vs. Active non-renal SLE** | | |
| 1^st^ | ALCAM | ALCAM |
| 2^nd^ | PF4 | PF4 |
| 3^rd^ | **-** | **-** |
| 4^th^ | **-** | **-** |
| **Accuracy** | 0.76 (0.71, 0.82) | 0.76 (0.71, 0.82) |
| **Balanced Accuracy** | 0.73 | 0.73 |
| **Brier** | 0.22 | 0.22 |
| **AUC** | 0.71 (0.64, 0.79) | 0.72 (0.65, 0.80) |
| **Sensitivity** | 0.76 | 0.76 |
| **Specificity** | 0.73 | 0.77 |
| **Active disease (active non-renal + active renal SLE) vs. Inactive SLE** | | |
| 1^st^ | ALCAM | ALCAM |
| 2^nd^ | - | - |
| 3^rd^ | - | - |
| 4^th^ | - | - |
| **Accuracy** | 0.64 (0.59, 0.70) | 0.64 (0.59, 0.70) |
| **Balanced Accuracy** | 0.71 | 0.71 |
| **Brier** | 0.19 | 0.18 |
| **AUC** | 0.72 (0.67, 0.78) | 0.74 (0.69, 0.79) |
| **Sensitivity** | 0.63 | 0.61 |
| **Specificity** | 0.80 | 0.80 |

**Supplementary Table 2:**

| Urine biomarker | ELISA cat. No. | Tested Dilutions | Optimal Dilution |
| --- | --- | --- | --- |
| ALCAM | R&D (DY656) | 1:2, 1:10, 1:50 | 1:2 |
| Cystatin-C | R&D (DY1196) | 1:2, 1:10, 1:50 | 1:50 |
| Hemopexin | IC (E-80HX) | 1:50 | 1:50 |
| KIM-1 | R&D (DY1750B) | 1:2, 1:10, 1:50 | 1:2 |
| Lipocalin2/NGAL | R&D (DY1757) | 1:5, 1:10, 1:50 | 1:5 |
| MCP-1 | R&D (DY279) | 1:5, 1:10, 1:50 | 1:5 |
| PF-4 | R&D (DY795) | 1:5, 1:10, 1:20 | 1:5 |
| Timp-1 | R&D (DY970) | 1:5, 1:10, 1:20 | 1:5 |
| TWEAK | R&D (DY1090) | 1:2, 1:5, 1:10 | 1:5 |
| VCAM-1 | R&D (DY809) | 1:10, 1:50, 1:100 | 1:100 |

IC: immunology consultant
